# The Blood Supply of the Human Pancreas: Anatomical and Surgical Considerations

**DOI:** 10.3390/jcm14165625

**Published:** 2025-08-08

**Authors:** George Triantafyllou, Orestis Lyros, Nikolaos Arkadopoulos, Panagiotis Kokoropoulos, Fotis Demetriou, Alexandros Samolis, Łukasz Olewnik, Ingrid C. Landfald, Maria Piagkou

**Affiliations:** 1 Department of Anatomy, School of Medicine, Faculty of Health Sciences, National and Kapodistrian University of Athens, 11527 Athens, Greece; georgerose406@gmail.com (G.T.); fotisdemetriou2000@gmail.com (F.D.); alexsamolis@me.com (A.S.); 2 “VARIANTIS” Research Laboratory, Department of Clinical Anatomy, Mazovian Academy in Plock, 09-400 Plock, Poland; lukaszolewnik@gmail.com (Ł.O.); ingridceciliee@gmail.com (I.C.L.); 3 Fourth Department of Surgery, Attikon University Hospital, School of Medicine, Faculty of Health Sciences, National and Kapodistrian University of Athens, 12462 Athens, Greece; lyrosorestis@gmail.com (O.L.); nakrado@hotmail.com (N.A.); kokoropoulos@yahoo.gr (P.K.); 4 Department of Clinical Anatomy, Mazovian Academy in Plock, 09-400 Plock, Poland

**Keywords:** pancreas, vasculature, pancreaticoduodenal artery, dorsal pancreatic artery, gastrocolic trunk, variation, pancreaticoduodenectomy

## Abstract

The pancreas exhibits a uniquely intricate vascular architecture characterized by frequent and clinically significant morphological variations. These variations—impacting both arterial supply and venous drainage—are critical determinants in surgical planning, radiologic interpretation, and interventional outcomes. This comprehensive review examines the full spectrum of pancreatic vascular anatomy, with particular emphasis on embryological development, imaging manifestations, and surgical relevance. Key arterial structures, including the superior and inferior pancreaticoduodenal arteries (SPDAs and IPDAs) and the dorsal pancreatic artery (DPA)—are explored in detail alongside accessory branches. On the venous side, focus is placed on the gastrocolic trunk (GCT) of Henle, the uncinate and centro-inferior pancreatic veins, and the dorsal pancreatic vein (DPV). The review highlights that arterial aberrations, such as a DPA originating from the superior mesenteric artery (SMA), or duplicated patterns of the IPDA, as well as venous anomalies such as variant drainage of the GCT or the centro-inferior pancreatic vein, have substantial implications during pancreaticoduodenectomy, distal pancreatectomy, and transplantation procedures. With advances in multidetector computed tomography (MDCT), magnetic resonance angiography (MRA), and three-dimensional (3D) modeling, high-risk vascular variants can now be accurately mapped preoperatively, facilitating safer and more effective minimally invasive and robotic-assisted surgeries. In conclusion, the recognition and understanding of pancreatic vascular variations are imperative for optimal surgical and interventional management. This review underscores the importance of multidisciplinary collaboration among surgeons, radiologists, and anatomists, which will allow them to integrate detailed anatomical knowledge into clinical workflows, ultimately improving patient outcomes in pancreatic procedures.

## 1. Introduction

The pancreas is one of the largest glands in the human body, serving both exocrine and endocrine functions. It secretes digestive enzymes and regulates blood glucose through hormone production. Anatomically, the pancreas is divided into the head, neck, body, tail, and uncinate process [1]. Positioned retroperitoneally, the pancreas lies horizontally across the posterior abdominal wall. The head lies within the duodenal loop, and the tail reaches the splenic hilum. The transverse mesocolon crosses its anterior surface, while posteriorly it is related to the abdominal aorta (AA), the origin of the superior mesenteric artery (SMA), the left suprarenal gland, the upper pole of the left kidney, and the left renal vein (LRV) [1].

The exocrine pancreas drains through the main pancreatic duct (MPD, of Wirsung) and the accessory duct (APD, of Santorini). The MPD typically runs along the central axis of the gland, while the APD drains the anterior part of the pancreatic head. Both ducts open into the major and minor duodenal papillae [1]. While the gross morphology of the pancreas is relatively consistent [2], the vascular and ductal architecture often exhibits significant variation, primarily due to its embryological development.

Embryologically, the pancreas arises from the foregut’s dorsal and ventral endodermal buds. The dorsal bud appears first and forms the head, neck, body, and tail. The ventral bud emerges near the hepatic diverticulum and gives rise to the lower part of the head and the uncinate process. Between the sixth and seventh weeks of gestation, rotation of the duodenum repositions the ventral bud posteriorly, allowing it to fuse with the dorsal bud. This fusion creates a unified organ with interconnected ductal and vascular networks [1,3]. Variants such as pancreas divisum, annular pancreas, or a dorsal pancreatic artery (DPA) originating from the SMA can be traced to deviations in this developmental process [2,4,5].

The arterial supply to the pancreas primarily arises from the celiac trunk (CeT) and the SMA, via the superior and inferior pancreaticoduodenal arteries (SPDA, IPDA), the DPA, and multiple small branches of the splenic artery (SA). Venous drainage occurs through the portal vein (PV), superior mesenteric vein (SMV), and splenic vein (SV), via tributaries including the superior and inferior pancreaticoduodenal veins (SPDV, IPDV) and the transverse pancreatic veins (TPVs). These vessels reflect the vascular territories of the embryonic foregut and midgut, accounting for many anatomical variants [3].

Numerous studies have examined these anatomical variants in detail, given the complex vasculature of the pancreas and its surgical importance, particularly in procedures such as pancreaticoduodenectomy. Vascular anomalies are common and may significantly impact surgical planning, risk of complications, and outcomes. As a result, a growing body of literature has focused on mapping and classifying these variants [6,7,8,9,10,11,12,13,14,15].

This review consolidates current knowledge on the vascular anatomy of the pancreas, with a focus on anatomical variations, their embryological origins, and their surgical relevance.

## 2. Arterial System of the Pancreas and Its Variations

### 2.1. Superior Pancreaticoduodenal Artery (SPDA)

The SPDA typically consists of two branches—anterior (aSPDA) and posterior (pSPDA)—both arising from the gastroduodenal artery (GDA), which itself originates from the common hepatic artery (CHA), a branch of the coeliac trunk (CeT). The aSPDA is usually the smaller branch of the GDA and the right gastroepiploic artery (GEA). It contributes to the anterior pancreaticoduodenal arcade through an anastomosis with the anterior branch of the inferior pancreaticoduodenal artery (aIPDA), supplying the anterior surfaces of the pancreatic head, uncinate process, and duodenum [1].

In contrast, the pSPDA is usually the first branch of the GDA, arising at the superior margin of the first portion of the duodenum. It anastomoses with the posterior branch of the IPDA (pIPDA) to form the posterior arcade, which vascularizes the posterior pancreatic head, duodenum, and distal bile duct. Notably, the pSPDA follows a spiral course—reflecting its embryological origin from the ventral pancreatic bud [1].

*Bergman’s Comprehensive Encyclopedia of Human Anatomic Variations* notes that the aSPDA is more consistent in origin than the pSPDA [2]. The aSPDA arises from the GDA in nearly all cases. However, rare origins from the SMA, replaced right hepatic artery (RRHA), CeT, proper hepatic artery (PHA), and SA have been reported. The pSPDA, although relatively consistent, exhibits greater variability. First misidentified as the “right SPDA” by Haller in 1745, its documented origins include the CHA, PHA, RHA (including accessory or replaced variants), LHA, SMA, DPA, and SA [2].

Despite its clinical importance, the SPDA has not been extensively studied. In an angiographic study, Toni et al. [16] visualized the SPDA in 94% of cases arising from the GDA. Bertelli et al. [17] identified multiple origin variants, including the CHA, SMA, CeT, SA, and PHA. The artery was absent in 1–2.5% of individuals and duplicated in 7.1% of cases. Studies that assessed the branches separately found similar patterns: Okahara et al. [18] confirmed that the aSPDA consistently originated from the GDA (100%) and noted anastomotic connections with the DPA in 14.2% of cases. Conversely, the pSPDA exhibited variant origins in 3.9% and anastomotic branches in 11.7% of cases. Comparable observations were made in angiographic studies by Macchi et al. [19] and in cadaveric dissections by Kumar et al. [20].

### 2.2. Inferior Pancreaticoduodenal Artery (IPDA)

The IPDA typically originates from the SMA at the lower border of the pancreas. It courses to the right, posterior to the superior mesenteric vein (SMV), and reaches the posterior surface of the uncinate process, where it bifurcates into the anterior (aIPDA) and posterior (pIPDA) branches. These arteries anastomose with their superior counterparts—the aSPDA and pSPDA—forming the anterior and posterior pancreaticoduodenal arcades, which supply the pancreatic head, uncinate process, and adjacent duodenum [1].

According to *Bergman’s Comprehensive Encyclopedia of Human Anatomic Variations*, the IPDA often arises from a common trunk with the first jejunal artery (JJA) of the SMA, forming what is known as the pancreaticoduodenojejunal trunk [2]. Less common origins include the accessory or replaced right hepatic artery, DPA, a common trunk with the third JJA, or the middle colic artery. Rarely, the IPDA may be duplicated—a finding initially described by Michels in 1955.

A systematic review and meta-analysis by Negoi et al. [21] reported the presence of the IPDA in 97.2% of cases. However, the artery’s origin showed variability in 20.2% of cases. The most frequent variant was a shared origin with the first jejunal artery, observed in 58.7% of cases.

Despite its importance, the IPDA has been inconsistently visualized across studies. Using angiography, Toni et al. [16] detected the artery in only 27% of scans. In contrast, Machi et al. [19] reported visualization in 53.8% of cases, and Okahara et al. [18] identified the IPDA in 77.5% of angiograms. Cadaveric dissections by Kumar et al. [20] revealed the vessel in 73.33% of specimens, suggesting a high anatomical variability or a high rate of absence.

While Toni et al. [16] found a 100% prevalence of SMA-origin IPDAs in their sample, Bertelli et al. [22] described multiple origin patterns. The most frequent was a shared trunk with the first jejunal artery, but other origins included the aberrant right hepatic, DPA, second or third jejunal arteries, and middle colic artery. Importantly, Tominaru et al. [23] noted that when the IPDA arises from the replaced right hepatic, its branching pattern differs significantly from the classic SMA origin—an essential consideration for surgical planning.

Infrequent variants have also been described. For example, Venieratos et al. [24] reported a case where the IPDA branched from a middle mesenteric artery—a rare vessel arising directly from the abdominal aorta.

### 2.3. Dorsal Pancreatic Artery (DPA)

The DPA typically originates from the SA, approximately 20 mm distal to its origin. It gives rise to several branches that anastomose with the pancreaticoduodenal arcades, while its terminal branch courses anterior or posterior to the SMV to supply the pancreatic head [1].

First described by Haller in 1745, the DPA is one of the most frequently studied arteries in the pancreas. Its presence varies across populations; it has been reported in 64% to 100% of individuals, with numerical variants ranging from a single DPA to multiple (up to four) vessels. Its origin is also variable, arising from the CeT, CHA, SMA, GDA, or, less commonly, from other branches [2].

A comprehensive meta-analysis by Rousek et al. [11], incorporating data from 30 studies, estimated the pooled prevalence of the DPA at 95.8%. The most common origin was the SA (37.6%), followed by the SMA (23.9%) and the CHA (18.3%). Other, less frequent sources included the CeT, aberrant hepatic arteries, GDA, middle colic artery, and even the IPDA. Multiple DPAs were documented in 40 cases across the reviewed literature [11].

While the DPA’s presence and origin are well described, its course, distribution, and branching patterns have received less attention. Its anatomical trajectory is influenced by its origin: when arising from the CeT, the DPA typically descends toward the pancreas; when originating from the SMA, it takes an ascending course [11]. The DPA predominantly supplies the distal pancreas, although branches to the pancreatic head have also been reported [25].

Through dissection studies, Yamane et al. [25] identified seven distinct branches from the DPA, which anastomosed with the SPDA, GDA, SA, and IPDA. In a later CT-based study, Yamane et al. [25] classified four major branch types: superior and inferior branches relative to the SV, a right dorsal branch to the PV supplying the uncinate process, and an accessory middle colic-like branch.

Rare variants have also been documented. For example, Di Gregorio et al. [26] reported an unusual case of the right GEA arising from the DPA, highlighting the complexity and variability of this vessel.

### 2.4. Other Pancreatic Branches

**Figure 1:**
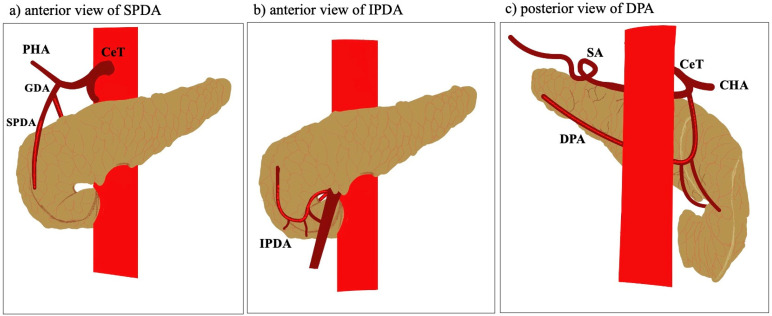
Schematic representation of the main pancreatic arteries. CeT—coeliac trunk; CHA—common hepatic artery; PHA—proper hepatic artery; GDA—gastroduodenal artery; SPDA—superior pancreaticoduodenal artery; IPDA—inferior pancreaticoduodenal artery; DPA—dorsal pancreatic artery; SA—splenic artery.

Beyond these main arteries (Figure 1), smaller and often unnamed branches can arise from the GDA, CHA, or even the right gastric and inferior phrenic arteries. Song et al. [27] highlighted the presence of pSPDA arising directly from hepatic arterial branches in a subset of patients. This finding emphasizes the complexity and redundancy of the pancreatic blood supply.

## 3. Venous System of the Pancreas and Its Variations

The venous drainage of the pancreas is intricate and exhibits considerable anatomical differences, both in the pancreatic head and the body–tail regions. Venous return primarily drains into the portal venous system via tributaries of the SMV and the SV, with numerous peripancreatic venous arcades and intrapancreatic tributaries contributing to this drainage [1].

### 3.1. Peripancreatic and Intrapancreatic Venous Anatomy

The anterior and posterior pancreaticoduodenal venous arcades drain the head of the pancreas. The posterior superior pancreaticoduodenal vein (pSPDV), the region’s most prominent and consistently visible vein, usually ascends between the common bile duct (CBD) and the duodenum to connect with the PV. Variations in its course include anterior (40%) and posterior (42%) relationships to the CBD, with rare cases of duplication or hypoplasia [28].

The anterior superior pancreaticoduodenal vein (aSPDV) typically drains into the GCT of Henle, a key convergence point for several veins. A systematic review found that Henle’s trunk is present in approximately 87% of people, typically comprising gastric, colic (superior right colic), and pancreatic (aSPDV) tributaries [29]. Variations in the aSPDV include duplication (17% of cases) and uncommon drainage patterns, such as direct entry into the SMV without forming a GCT [28].

The posterior inferior (pIPDV) and anterior inferior pancreaticoduodenal veins (aIPDV) often merge to form the inferior pancreaticoduodenal vein (IPDV), which typically drains into the first jejunal vein. However, various configurations of these tributaries and their terminations are common, highlighting their complementary role alongside the uncinate and direct draining veins [28].

Small intrapancreatic veins in the pancreatic head often drain directly into the portal vein or SMV. These veins are usually short and oriented toward the center, and they were seen in 94% of individuals using multidetector CT imaging. The uncinate vein, which appears in about 30% of cases, runs behind the medial side of the pancreatic head to drain into the posterior wall of the PV [28,30].

### 3.2. Body and Tail Venous Drainage

**Figure 2:**
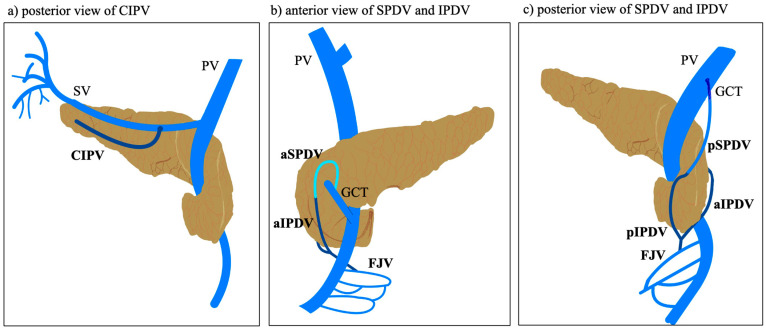
Schematic representation of the main pancreatic veins. PV—portal vein; SV—splenic vein; CIPV—centroinferior pancreatic vein; GCT—gastrocolic trunk; aSPDV—anterior superior pancreaticoduodenal vein; aIPDV—anterior inferior pancreaticoduodenal vein; FJV—first jejunal vein; pSPDV—posterior superior pancreaticoduodenal vein; pIPDV—posterior inferior pancreaticoduodenal vein.

Venous return from the pancreatic body and tail can also vary quite a bit. The centro-inferior pancreatic vein (CIPV), which runs along the lower edge of the body, is always identified and may drain into the SV (59%), SMV (40%), or inferior mesenteric vein (IMV) (30%). In all cases, multiple small veins that drain directly into the SV were observed, usually arching beneath the tissue or passing through the center of the pancreatic body and tail [28,30,31]. The dorsal pancreatic vein (DPV), a less common structure, runs from the back of the pancreatic neck to the SV, or, less often, the SMV. Its presence was noted in only 4% of cases [28] (Figure 2).

## 4. Surgical Importance of Pancreatic Vascular Variations

The vascular anatomy of the pancreas is a complex network that often varies from textbook descriptions. These arterial and venous variations, while typically asymptomatic in normal conditions, become critically important during pancreatic and peri-pancreatic surgeries. Recognizing these variations is crucial for planning and performing procedures such as pancreaticoduodenectomy, total or distal pancreatectomy, spleen-preserving pancreatectomy, and complex oncologic resections involving the liver, duodenum, and colon.

Arterial anomalies can affect surgical safety, influence the decision to resect or preserve vessels, and impact postoperative pancreatic perfusion. For instance, anterior and posterior pancreaticoduodenal arcades are commonly encountered during pancreaticoduodenectomy. A variant origin of the pSPDA from the SMA or right hepatic artery may not be expected, and if injured, can lead to hepatic ischemia, especially in patients with other variants in the hepatic arterial system. Intraoperative bleeding from the SPDA or its branches is often severe due to the high-pressure flow and retroperitoneal location, making hemostasis challenging and increasing operative time [32,33].

The DPA plays a crucial role in central pancreatectomy, which is gaining popularity for benign or borderline malignant lesions of the pancreatic neck [11]. It supplies segmental blood flow to the body and neck; inadvertent ligation without compensation from the SPDA or SA branches may lead to ischemia of the remaining tissue. Variant DPA origins from the SMA, CeT, or middle colic artery further complicate dissection and require detailed preoperative imaging [11].

For spleen-preserving distal pancreatectomy (*Warshaw technique*), the SA is typically preserved to maintain blood flow to the spleen via the short gastric and left GEA. Variations of the DPA that originate from non-splenic sources or duplicated DPAs must be identified to prevent splenic infarction or segmental necrosis of the pancreatic tail [11]. From an interventional radiology perspective, arterial variations can impact the success of embolization procedures in cases of pancreatic bleeding, pseudoaneurysm, or arterial steal syndrome following a transplant. For instance, transcatheter embolization of a bleeding DPA pseudoaneurysm requires careful mapping of its origin and collateral pathways to avoid non-target embolization [11].

Venous variants are arguably more critical during surgery because of their thin walls, lack of muscular support, and proximity to major blood vessels. Inadvertent injury can lead to uncontrollable bleeding and PV thrombosis, potentially requiring urgent vascular reconstruction.

The GCT of Henle, due to its variable anatomy and the confluence of multiple tributaries, is a crucial structure during right hemicolectomy, pancreaticoduodenectomy, and mesocolic dissections. Its injury can cause significant bleeding, especially in laparoscopic or robotic-assisted surgeries where field exposure is limited. Additionally, ligation of the GTH without understanding its tributaries may impair venous drainage from the right colon or anterior pancreas, leading to congestion or venous infarction [34,35].

During the uncinate process dissection in a *Whipple procedure*, the uncinate vein and small posterior veins draining into the SMV or PV must be carefully ligated. These vessels are often located behind the pancreas and in front of the SMA. Injury to these veins without proper control can cloud the surgical view and increase the risk of iatrogenic SMA injury [36]. In distal pancreatectomy, the CIPV and small veins draining the body and tail should be preserved if possible. If inadvertently ligated or missed, there is a risk of segmental venous congestion, which may lead to pancreatic fistula, especially if drainage of the remaining pancreas is blocked. Although rare, the DPV can be a critical collateral between the SV and SMV systems. Recognizing it is vital in patients with portal hypertension, where it may enlarge and cause varices or intraoperative bleeding [33,34].

In oncologic surgery, vascular involvement often determines resectability criteria. For pancreatic head cancers, invasion of the SMV-PV confluence may require venous resection and reconstruction. Variant veins, such as a duplicated pSPDV or CIPV draining into the SMV or IMV, can serve as alternative venous return pathways after resection and should be preserved if possible. Preoperative venous mapping aids in planning resection margins, anastomotic options, and assessing the need for grafts or autologous vein interposition [32,35].

Understanding pancreatic venous outflow is crucial in pancreas transplantation, especially with segmental pancreas grafts or islet cell transplantation. The DPV, CIPV, and small parenchymal veins must be carefully reconstructed or assessed for graft perfusion. Disregarding variations in venous outflow can result in graft thrombosis or ischemia–reperfusion injury.

The significance of detailed vascular imaging cannot be overstated. Triphasic contrast-enhanced MDCT, MRA, and intraoperative Doppler ultrasonography (US) are standard techniques for mapping vascular anatomy. Three-dimensional reconstruction and volume-rendering methods enable virtual surgical planning and are routinely used in high-volume centers. Incorporating vascular mapping into navigation systems for robotic-assisted surgery is a growing trend that can help reduce the risk of vascular injury.

The mesopancreas was first described as the plate-like structure between the pancreatic head and the CeT/SMA and it is considered an important anatomical entity for major oncological benefit [37,38,39]. Recent anatomical insights further elucidate this anatomical region, emphasizing the role of fasciae—such as the retropancreatic fascia of Treitz and the P-A ligament (pancreas–aorta/CeT/SMA ligament)—in defining vascular and neural dissection planes. These structures contain the mesopancreas, a crucial site for lymphovascular spread and recurrence, which is central to total mesopancreas excision concepts [37,38,39]. Recognizing the fascia as a dissection boundary reduces bleeding by maintaining clear surgical planes. These findings support the use of fascia-guided surgery, particularly in robotic and laparoscopic techniques, where enhanced visualization facilitates the identification of avascular planes [39].

The development of pancreatic surgery has quickly transformed from open procedures to minimally invasive and robotic-assisted methods, driven by the goal of reducing complications, shortening recovery times, and increasing precision. However, this progress has highlighted the need for detailed vascular knowledge, as limited tactile feedback and restricted visual fields can increase the risk due to anatomical variations. Incorporating detailed vascular mapping into robotic systems and laparoscopic procedures is now considered an essential component of modern pancreatic surgery [40,41,42,43,44,45].

For example, recent advancements in complex pancreatic resections—particularly distal pancreatectomy with en bloc celiac axis resection—highlight the importance of preoperative arterial mapping and intraoperative vascular control [45]. While the procedure offers oncologic benefits, it must be performed with careful attention to arterial flow redistribution. Preserving the pancreaticoduodenal arcades is crucial when resecting the celiac axis. Liu et al. [45] emphasize that the procedure is associated with longer operative times, increased postoperative mortality, and extended hospital stays compared to standard distal pancreatectomy. They also stress that confirming an intact collateral route via the SMA through the pancreatic head’s arcade intraoperatively—often using Doppler ultrasound (DUS)—is essential to ensure ongoing hepatic perfusion. Understanding and identifying these collateral pathways are vital for procedural success and postoperative organ viability [45].

In robotic-assisted pancreaticoduodenectomy, vascular variations such as an aberrant right hepatic, ventrally positioned first jejunal vein, or different origins of the IPDA can significantly affect the feasibility and safety of the artery-first approach. The anterior SMA-first dissection requires precise identification and ligation of the IPDA and its arcades without impairing hepatic or intestinal blood flow. Preoperative 3D vascular reconstruction using MDCT or MRA has proven essential in guiding these critical steps, providing a “surgical roadmap” for safe vascular control [35,36].

Robotic systems equipped with enhanced magnification and articulated instrumentation allow for precise dissection around variant vessels, including the GCT of Henle and the uncinate vein plexus. However, this advantage depends on preoperative anticipation of such variations. For example, Kang et al. [34] reported that unrecognized duplication of the pSPDV or abnormal drainage into the SMV increased intraoperative bleeding risk during robotic pancreaticoduodenectomy. Similarly, recognizing a duplicated or dominant DPA is essential in central pancreatectomy to prevent ischemia of the pancreatic remnant. Zhang et al. [46] emphasize that total laparoscopic pancreaticoduodenectomy, despite being technically challenging, results in less blood loss, shorter ICU stays, and higher R0 resection rates than open procedures. Nevertheless, these benefits rely on thorough preoperative vascular planning, especially in cases with duplicated veins or abnormal IPDA origins that could be missed intraoperatively [46].

Whether performed laparoscopically or robotically, minimally invasive distal pancreatectomy poses particular challenges in the setting of variant SA branches or accessory IPVs [47]. Preservation of vessels such as the CIPV is often more technically demanding in laparoscopy due to limited mobilization angles and proximity to the splenic hilum. Robotic assistance offers superior dexterity for vessel preservation or precise clip placement, particularly in spleen-preserving techniques such as the Warshaw procedure. According to Lyu et al. [48] while laparoscopic distal pancreatectomy offers advantages such as lower estimated blood loss, fewer complications, and a shorter hospital stay compared to open surgery, its safety hinges on precise vascular dissection. In centers with lower operative volumes or inadequate imaging resources, failure to recognize variant anatomy can increase the incidence of postoperative pancreatic fistula and hemorrhage [48].

Thus, the collaboration between vascular anatomy, preoperative imaging, and robotic technology illustrates a new era of precise pancreas surgery. Mastery of anatomical variations is no longer limited to anatomists or radiologists—it is now essential for every robotic and minimally invasive pancreatic surgeon.

**Figure 3:**
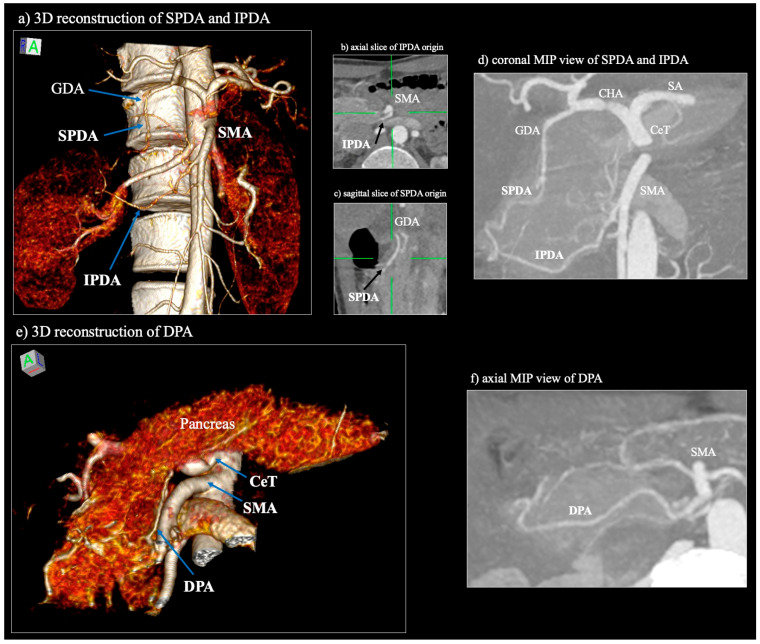
Example of the computed tomography angiography of a 42-year-old female patient depicting the main pancreatic arteries. CeT—coeliac trunk; SMA—superior mesenteric artery; GDA—gastroduodenal artery; CHA—common hepatic artery; SPDA—superior pancreaticoduodenal artery; IPDA—inferior pancreaticoduodenal artery; DPA—dorsal pancreatic artery; SA—splenic artery.

Accurate identification of pancreatic vascular variants is critical in minimally invasive and image-guided surgery. Modern imaging technologies, such as triphasic contrast-enhanced MDCT, MRA, and DSA, offer high spatial resolution, enabling detailed visualization of both major and minor vascular structures (Figure 3).

Triphasic MDCT, particularly in arterial and venous phases, is the primary modality for vascular mapping. It consistently achieves over 90% sensitivity in detecting major arterial trunks, including the SPDA, IPDA, and DPA [11,25]. Additionally, 3D reconstructions and volume-rendered imaging enhance preoperative planning by clarifying vessel trajectories, branching patterns, and surgically relevant anomalies.

CTA is particularly effective in detecting rare variations, such as duplicated DPAs, aberrant origins of the IPDA, and accessory arterial connections between the splenic and mesenteric territories. For instance, Yamane et al. [25] used contrast-enhanced CT to classify DPA branching patterns into superior, inferior, right, and collateral types—each with direct implications for procedures like central pancreatectomy or mesopancreas dissection. Inadvertent ligation of key branches during these surgeries may result in ischemia to the remaining pancreatic tissue. Venous phase imaging is crucial for visualizing the gastrocolic trunk of Henle and its key tributaries, including the aSPDV, right gastroepiploic vein, and superior right colic vein. These insights are especially critical in surgeries like pancreaticoduodenectomy and right hemicolectomy, where compromised venous outflow can lead to significant postoperative complications. Dynamic imaging also helps differentiate between compressible or thrombosed variant veins and tumor infiltration or enlarged lymph nodes, which may alter resection margins or lymphadenectomy planes [35,36].

Although MRA is less commonly used for routine evaluation, it remains valuable in patients with contrast allergies or renal impairment, as it is less prone to motion artifacts and offers higher resolution for small-caliber vessels. Intraoperative DUS also plays a crucial role in real-time perfusion assessment, particularly during trial clamping of arteries such as the GDA or IPDA. Integrating radiologic expertise into surgical planning is now a cornerstone of high-quality pancreatic care. Structured reporting of vascular anomalies, 3D modeling for preoperative simulation, and augmented reality tools define the future of pancreas surgery, where imaging is no longer just diagnostic, but decisively operative.

## 5. Conclusions

The pancreas has a highly intricate vascular network with significant variations in arterial supply and venous drainage. An important summary of the present comprehensive review is presented in Table 1. While often asymptomatic, these variations become crucial during pancreatic surgeries and interventional procedures. This review highlights key patterns and rare anomalies involving the SPDA, the IPDA, the DPA, and venous structures, including the GCT of Henle, the uncinate and CIPVs, and the DPV. These vascular variants are not merely academic curiosities; they have a direct impact on surgical safety and clinical outcomes. If unrecognized intraoperatively, arterial anomalies can alter perfusion, necessitate complex reconstructions, or cause unexpected ischemia. Venous variations, due to their fragility and variability, pose risks of severe bleeding, congestion, or thrombosis, especially during complex procedures like pancreaticoduodenectomy. Vascular anatomy influences resectability and guides decisions on the extent of vascular resection in oncologic surgery. In transplant and interventional radiology, understanding these variations is essential for graft planning, embolization, and bypass strategies. Thanks to advanced MDCT, MRA, and 3D modeling, the ability to identify and map these variations has become fundamental to modern surgical practice. A precise understanding of pancreatic vascular anatomy is indispensable for safe and effective surgical planning. Collaboration between surgeons, radiologists, and anatomists is essential to document, share, and apply this knowledge. As imaging and surgical technology evolve, anatomical accuracy will remain central to improving outcomes and reducing complications in pancreatic surgery.

## Figures and Tables

**Table 1:** Origins and patterns of key pancreatic vessels, DPA (dorsal pancreatic artery), SPDA (superior pancreaticoduodenal artery), IPDA (inferior pancreaticoduodenal artery), and CIPV (centro-inferior pancreatic vein).

Feature	DPA	SPDA	IPDA	CIPV
Typical Origin	Splenic artery (SA) [1]	Gastroduodenal artery (GDA) [1]	Superior mesenteric artery (SMA) [1]	Pancreatic body/tail venous plexus [1]
Branches/Subtypes	Superior, inferior, right dorsal [1]	Anterior (aSPDA) and posterior (pSPDA) [1]	Anterior (aIPDA) and posterior (pIPDA) [1]	Multiple small veins; no named sub-branches [1]
Embryologic Correlation	Foregut vasculature (often dorsal bud) [3]	Ventral pancreatic bud (especially pSPDA) [3]	Midgut vasculature (uncinate from ventral bud) [3]	Drains the parenchymal blood from the lower pancreas [3]
Clinical Significance	Key in central pancreatectomy; high variation risk [11]	Commonly involved in pancreatoduodenectomy; hemorrhage risk [32,33]	Crucial in SMA—first dissection during Whipple; variant trunks complicate dissection [32,33]	Must be preserved in distal pancreatectomy to avoid segmental congestion [33,34,36]
Surgical Relevance	Needs identification in the Warshaw technique and central pancreatectomy; ischemia risk if sacrificed [11]	Ligation may cause hepatic ischemia if the origin is aberrant [32,33]	Collateral flow is critical when the celiac axis is resected; multiple trunks are possible [32,33]	Misidentification can lead to postoperative pancreatic fistula or venous infarction [33,34,36]
Imaging Visibility	MDCT + CTA: ~95% sensitivity; branching types visualized on 3DCT [25]	Easily visualized in most CTAs; duplication in 7.1% cases [32,33]	Often missed: visualized in only 27–77% of angiograms [32,33]	Seen in 100% of cases with careful venous-phase CT [33,34,36]
Variability	Extremely high; duplicated in ~10–12%, multiple origins [11,25]	pSPDA is more variable than aSPDA; rare origins from SMA or SA [17,22]	Common trunk with JJA in ~58.7%; origin varies in ~20.2% [17,22]	Highly variable; drainage site differs among SMV, SV, and IMV [33,34,36]
Risk if Unrecognized	Segmental ischemia; failed reconstruction in transplant or resections [11,25]	Retroperitoneal hemorrhage; unexpected hepatic ischemia [32,33]	Misidentification may lead to injury of the SMA or jejunal branches [32,33]	Venous congestion, hemorrhage, or graft thrombosis risk in surgery or transplant [33,34,36]

## Data Availability

All the data are available upon reasonable request to the corresponding author.

## Abbreviations

The following abbreviations are used in this manuscript:

aIPDV	Anterior Inferior Pancreaticoduodenal Vein.
APD	Accessory Pancreatic Duct.
aIPDA	Anterior Inferior Pancreaticoduodenal Artery.
aSPDA	Anterior Superior Pancreaticoduodenal Artery.
aSPDV	Anterior Superior Pancreaticoduodenal Vein.
CBD	Common Bile Duct.
CeT	Celiac Trunk.
CHA	Common Hepatic Artery.
CIPV	Centro-Inferior Pancreatic Vein.
CT	Computed Tomography.
DPA	Dorsal Pancreatic Artery.
DSA	Digital Subtraction Angiography.
GDA	Gastroduodenal Artery.
GTH	Gastrocolic Trunk of Henle.
IMV	Inferior Mesenteric Vein.
IPDA	Inferior Pancreaticoduodenal Artery.
IPDV	Inferior Pancreaticoduodenal Vein.
MRA	Magnetic Resonance Angiography.
MPD	Main Pancreatic Duct.
MDCT	Multidetector Computed Tomography.
pIPDV	Posterior Inferior Pancreaticoduodenal Vein.
pIPDA	Posterior Inferior Pancreaticoduodenal Artery.
pSPDA	Posterior Superior Pancreaticoduodenal Artery.
pSPDV	Posterior Superior Pancreaticoduodenal Vein.
PV	Portal Vein.
SA	Splenic Artery.
SMA	Superior Mesenteric Artery.
SMV	Superior Mesenteric Vein.
SPDA	Superior Pancreaticoduodenal Artery.
SPDV	Superior Pancreaticoduodenal Vein.
SV	Splenic Vein.
TPV	Transverse Pancreatic Vein.
